# A Comparison of Classical and H-Type Bovine Spongiform Encephalopathy Associated with E211K Prion Protein Polymorphism in Wild-Type and EK211 Cattle Following Intracranial Inoculation

**DOI:** 10.3389/fvets.2016.00078

**Published:** 2016-09-15

**Authors:** S. Jo Moore, M. Heather West Greenlee, Jodi D. Smith, Catherine E. Vrentas, Eric M. Nicholson, Justin J. Greenlee

**Affiliations:** ^1^Virus and Prion Research Unit, Agricultural Research Service, National Animal Disease Center, United States Department of Agriculture, Ames, IA, USA; ^2^Department of Biomedical Sciences and Interdepartmental Toxicology Program, Iowa State University, Ames, IA, USA

**Keywords:** bovine spongiform encephalopathy, brain diseases, cattle, E211K, prion protein, transmissible spongiform encephalopathy

## Abstract

In 2006, a case of H-type bovine spongiform encephalopathy (BSE-H) was diagnosed in a cow that was associated with a heritable polymorphism in the bovine prion protein gene (*PRNP*) resulting in a lysine for glutamate amino acid substitution at codon 211 (called E211K) of the prion protein. Although the prevalence of this polymorphism is low, cattle carrying the K211 allele may be predisposed to rapid onset of BSE-H when exposed or to the potential development of a genetic BSE. This study was conducted to better understand the relationship between the K211 polymorphism and its effect on BSE phenotype. BSE-H from the US 2006 case was inoculated intracranially (IC) in one *PRNP* wild-type (EE211) calf and one EK211 calf. In addition, one wild-type calf and one EK211 calf were inoculated IC with brain homogenate from a US 2003 classical BSE case. All cattle developed clinical disease. The survival time of the E211K BSE-H inoculated EK211 calf (10 months) was shorter than the wild-type calf (18 months). This genotype effect was not observed in classical BSE inoculated cattle (both 26 months). Significant changes in retinal function were observed in H-type BSE challenged cattle only. Cattle challenged with the same inoculum showed similar severity and neuroanatomical distribution of vacuolation and disease-associated prion protein deposition in the brain, though differences in neuropathology were observed between E211K BSE-H and classical BSE inoculated animals. Western blot results for brain tissue from challenged animals were consistent with the inoculum strains. This study demonstrates that the phenotype of E211K BSE-H remains stable when transmitted to cattle without the K211 polymorphism, and exhibits a number of features that differ from classical BSE in both wild-type and heterozygous EK211 animals.

## Introduction

The transmissible spongiform encephalopathies (TSEs) are a group of fatal neurological diseases associated with the post-translational conversion of the cellular form of the prion protein (PrP^C^) to a more protease-resistant isoform (PrP^Sc^) (1, 2). Prion protein gene (*PRNP*) polymorphisms can alter the amino acid sequence and, therefore, the post-translational folding properties of PrP^C^, leading to variable susceptibility to infection or spontaneous emergence of prion disease. A number of disease-associated *PRNP* polymorphisms have been described for human prion diseases. Of these, codon 129 has a strong influence on susceptibility to acquired, sporadic, and familial prion disease, and the E200K mutation is the most frequent *PRNP* mutation associated with familial Creutzfeldt–Jakob disease (fCJD) (3, 4).

Bovine spongiform encephalopathy (BSE) was first identified in the late 1980s in cattle in the United Kingdom (5). Subsequent epidemiological investigations suggested transmission through exposure to feedstuffs contaminated with infectious ruminant-derived protein (6, 7). In 2006, a case of H-type BSE (BSE-H) was detected in the United States (U.S.) in an animal with a novel polymorphism in *PRNP* associated with a substitution of lysine for glutamate at codon 211 (E211K) (8). It was subsequently discovered that the only living offspring of this cow also carried the E211K polymorphism, demonstrating that the allele is heritable and homologous to the E200K mutation in sporadic/genetic Creutzfeldt–Jakob disease (9). Intracranial challenge of an EK211 calf with brain homogenate from the 2006 U.S. BSE-H case (i.e., its granddam) resulted in rapid development of BSE with clinical and pathological features consistent with BSE-H (10).

Bovine spongiform encephalopathy in cattle can be differentiated into three types based on the apparent molecular mass of the three prion protein fragments visualized on a western immunoblot. In classical BSE (BSE-C), the BSE type associated with the majority of BSE cases, including those in the United Kingdom BSE epizootic, the non-glycosylated PrP^Sc^ band migrates at ~17 kDa. In atypical BSEs, the non-glycosylated PrP^Sc^ band runs 0.5 kDa lower in low-type BSE (BSE-L) or 1–2 kDa higher in high-type BSE (BSE-H), when compared to BSE-C (11–15). In addition to differences in western blot migration pattern, the epidemiology of classical and atypical BSE cases are different. Most cases of atypical BSE occur in animals 8 years and older (16) while the risk of detection of C-BSE is highest at 5–6 years of age (17–19). Atypical BSE cases occur at a very low prevalence, have been reported worldwide, including in countries that are free of BSE-C, and there is a lack of epidemiologic links to other TSEs, leading to the hypothesis that they are sporadic diseases (20).

The purpose of this study was to investigate the effect of the K211 polymorphism on BSE phenotype. To test this, brain homogenate from the U.S. 2006 H-type BSE case bearing the E211K polymorphism was inoculated into wild-type (EE211) calves and EK211 calves. Genotype matched animals were inoculated with BSE-C to test whether the K211 allele could affect the molecular profile and mask the appearance of feedborne BSE-C.

## Materials and Methods

### Ethics Statement

This experiment was carried out in accordance with the *Guide for the Care and Use of Laboratory Animals, 8th edition* (Institute of Laboratory Animal Resources, National Academy of Sciences, WA, USA) and the *Guide for the Care and Use of Agricultural Animals in Research and Teaching* (Federation of Animal Science Societies, Champaign, IL, USA). The protocol was approved by the Institutional Animal Care and Use Committee at the National Animal Disease Center (protocol number: 3985).

### Animals and Procedures

Two EK211 calves and two wild-type (EE211) calves were generated by embryo transfer from the only known offspring of the US 2006 atypical BSE case (9). Genotypes were confirmed by sequencing of the *PRNP* (9).

The BSE-H inoculum was prepared from brain homogenate from the brainstem of the U.S. 2006 atypical BSE case possessing the E211K *PRNP* polymorphism. The BSE-C inoculum was prepared from brain homogenate from a U.S. classical BSE case that was diagnosed in 2003 (21).

At ~2 months of age one EK211 calf and one wild-type calf were inoculated intracranially (IC) with 1 ml of a 10% (w/v) homogenate of the BSE-H inoculum. In addition, one EK211 calf and one wild-type calf were inoculated IC with 1 ml of a 10% (w/v) homogenate of the BSE-C inoculum.

Intracranial inoculations were performed as described previously (10). Briefly, each calf was sedated with xylazine, the frontal area was clipped and scrubbed, a 1 cm midline incision was made in the skin slightly caudal to the junction of the parietal and frontal bones, and a 1 mm hole was drilled through the calvarium. A 22-gage spinal needle was advanced through the hole perpendicular to the frontal bones until the tip of the needle made contact with the opposite (bottom) side of the calvarium. The inoculum was slowly injected as the needle was withdrawn through the brain. The skin was closed with tissue glue (Vetbond, 3M, St. Paul, MN, USA).

Calves were observed daily for clinical signs of disease and euthanized when unequivocal signs of TSE (see Results) were noted. Two sets of tissue samples were collected comprising representative sections of liver, kidney, spleen, skin, striated muscles (heart, tongue, diaphragm, and masseter), thyroid gland, turbinates, trachea, lung, esophagus, rumen, reticulum, omasum, abomasum, small intestine, including ileum, adrenal gland, pancreas, urinary bladder, lymph nodes (retropharyngeal, prescapular, mesenteric, and popliteal), tonsils (palatine and nasopharyngeal), nerves (sciatic, optic, and trigeminal), pituitary gland, trigeminal ganglion, brain (cerebral cortex, cerebellum, midbrain, including superior colliculus, brainstem including obex), spinal cord (cervical, thoracic, lumbar), and eye were collected.

The first set was collected into 10% buffered formalin (globes were fixed in Bouin’s fixative), embedded in paraffin wax, and sectioned at 5 μm for staining with hematoxylin and eosin (HE) and immunolabeling with anti-prion protein antibodies. The second set of tissues was frozen.

### Electroretinography

Electroretinography was performed on challenged cattle as previously described (22) prior to inoculation, and at 6, 9, and 12 months post inoculation (MPI). An EPIC 4000 visual electrodiagnostic testing system (LKC Technologies, Gaithersburg, MD, USA) with a CMGS-1 Color Mini-Ganzfeld Stimulator (LKC Technologies, Gaithersburg, MD, USA) was used to capture electroretinograms (ERG). The left eye was tested at each time point. The animals were dark adapted for 20 min, followed by a scotopic recording (single white flash 2.45 cd s/m^2^, three technical replicates). Electroretinography was also performed on age-matched, genotype matched, non-inoculated control animals (*n* = 2 EE211, *n* = 2 EK211) to provide negative control values. Statistical analysis of ERGs was performed with GraphPad Prism 6. Paired *t*-tests were used to compare ERG values across time points for each animal.

### Optical Coherence Tomography

Retinas were imaged 1 day prior to inoculation (0 MPI), at 12 MPI, and prior to necropsy when animals displayed unequivocal signs of clinical disease. A Bioptigen SD-OCT (Bioptigen, Durham, NC, USA) was used to capture linear B scans (6 mm; 1000 A scans/B scan). Scans were taken from the central retina. At each time point at least 10 measurements per animal of retinal thickness were taken from multiple scan frames (using on-screen calipers) to determine an average thickness measurement for each animal. The values reported are ±standard deviation of the measurements. Retinal thickness was also measured for aged matched, genotype matched, non-inoculated control animals (*n* = 2 EE211, *n* = 2 EK211) to provide negative control values. Statistical analysis of optical coherence tomography (OCT) data was performed with GraphPad Prism 6. OCT data were grouped by time point and analyzed for differences between groups using the Mann–Whitney test.

### Histopathology

Vacuolation profiles were generated by examining 17 defined regions of brain on HE stained sections and scoring the severity of vacuolation as described previously (23). Neuroanatomical areas examined were as follows: nucleus of the solitary tract, nucleus of the spinal tract of the trigeminal nerve, hypoglossal nucleus, vestibular nuclear complex, cochlear nucleus, cerebellar vermis, central gray matter, rostral colliculus, medial geniculate nucleus, hypothalamus, nucleus dorsomedialis thalami, nucleus ventralis lateralis thalami, frontal cortex, septal nuclei, caudate nucleus, putamen, and claustrum.

### Immunohistochemistry

Immunohistochemistry was used to assess accumulation of PrP^Sc^ and, in the eye, the resulting response of retinal tissue.

Tissue sections from non-central nervous system tissues were immunostained by an automated immunohistochemical method for detection of PrP^Sc^ as described previously (10). Briefly, after deparaffinization and rehydration, tissue sections were autoclaved for 30 min in an antigen retrieval solution (DAKO Target Retrieval Solution, DAKO Corp., Carpinteria, CA, USA) and stained with an indirect, biotin-free staining system containing an alkaline phosphatase labeled secondary antibody (ultraview Universal Alkaline Phosphatase Red Detection Kit, Ventana Medical Systems, Inc., Tucson, AZ, USA) designed for an automated immunostainer (NexES IHC module, Ventana Medical Systems, Inc., Tucson, AZ, USA). The primary antibody used was F99/97.6.1 [O’Rourke et al. (24), Pullman, WA, USA] at a concentration of 10 μg/ml, and incubation was carried out at 37°C for 32 min. Slides were counterstained with Gill’s hematoxylin and bluing agent (Ventana Medical Systems, Tucson, AZ, USA) then coverslipped.

Tissues sections from the brain, spinal cord, and eye were immunostained manually. After deparaffinization and rehydration, tissue sections were autoclaved at 100°C for 20 min in an antigen retrieval solution (Dako Target Retrieval Solution, Dako Corp., Carpinteria, CA, USA). Tissue sections were cooled, then washed, then immersed in methanol containing 0.3% H_2_O_2_ for 20 min. Tissue sections were immunostained for PrP^Sc^ using the primary antibodies F99/97.6.1 at a concentration of 2 μg/ml, 6C2 [Rigter et al. (25), Central Veterinary Institute, Lelystad, Netherlands] at a concentration of 1 μg/ml, 12B2 [Langeveld et al. (26), Central Veterinary Institute, Lelystad, Netherlands], or R145 (eye only) (27) at a concentration of 0.2 μg/ml. Incubation was carried out at 4°C overnight. A proprietary polymer (Dako polymer, Dako Corp., Carpinteria, CA, USA) linked to HRP was added for 10 min. The reaction was visualized using 3,3′-diaminobenzidine tetrachloride (DAB 2, Vector Laboratories, Burlingame, CA, USA) as the chromagen for 10 min. Slides were counterstained with hematoxylin, rehydrated, then coverslipped.

In the eye, immunoreactivity for rabbit anti-GFAP (DAKO, diluted at 1:7500) was visualized with an EnVision HRP-System as directed (DAKO). Images for the figures were captured using a Nikon DS camera on a Nikon Eclipse 50i microscope.

PrP^Sc^ profiles were generated by scoring the severity of defined morphological immunolabeling types in 10 brain regions at the level of the basal nuclei as described previously (28). Histological sections immunolabeled with the anti-prion protein antibody 6C2 were used for this examination.

### Molecular Characterization

Western blotting on the BSE samples was performed as described previously (10). Briefly, brain homogenate was prepared in a 1× homogenization buffer (Prionics AG, Switzerland) and digested with proteinase K at 37°C for 1 h. Samples were diluted 1:4 in 4× SDS-PAGE sample buffer and analyzed by standard western blotting procedures. Western blot detection was conducted using mouse anti-PrP monoclonal antibodies (mAb) 6H4 at a 10:000 dilution (0.1 μg/ml), P4 at a 1:10,000 dilution (0.1 μg/ml), or SAF-84 at 1:200 dilution (1 μg/ml) as the primary antibody.

Deglycosylation of PrP^res^ was performed as described previously (10). Briefly, 5–10 μl of 10% brain homogenate was PK digested as above and denatured in sodium dodecyl sulfate (final concentration 3% w/v) at 100°C for 10 min. The PK-digested, denatured homogenates (5–10 μl) were incubated with PNGase F (final concentration 150 U/μl) at 37°C for ≥16 h. The samples were combined with loading buffer and heated to 100°C for 5 min prior to gel electrophoresis.

### Glycoprofile Generation

The glycoprofile is based on the relative proportions of the unglycosylated, monoglycosylated, and diglycosylated PrP^Sc^ bands visible on western blot. PrP^Sc^ signals were analyzed with image-analysis software (ImageJ, National Institutes of Health) using seven technical replicates of the same brain homogenate per animal.

### IDEXX ELISA-Based PrP^Sc^ Stability Assays

Determination of PrP^Sc^ stability by the use of the IDEXX HerdChek BSE ELISA (in the absence of proteinase K digestion) was performed as described previously (29) with modifications to improve buffering with high concentrations of brain homogenate (30). Briefly, whole homogenates (lacking PK treatment) were prepared by beadbeating in Dulbecco’s PBS with 10X buffering strength, and homogenate samples were incubated with increasing concentrations of guanidine hydrochloride (GdnHCl; 0.25–4 or 0.25–5 M) for 1 h in 10× PBS. The level of PrP^Sc^ remaining at each (GdnHCl) was assessed immediately after sample dilution to 0.25 M GdnHCl by application to the HerdChek BSE ELISA plate. Values at each (GdnHCl) were normalized to the signal for a 0.25 M GdnHCl treatment point. Control samples included PrP^Sc^ from *n* = 3 non-E211K BSE-H cases [cases #5–7 in Table 1 of (30)] and PrP^Sc^ from *n* = 4 BSE-C-infected cases [cases #1–4 in Table 1 of Ref. (30)]. Comparison of stability curves was based on the (GdnHCl)_1/2_ value, i.e., the concentration of GdnHCl required to reduce the level of the PrP^Sc^ signal by half compared to the 0.25 M normalization point.

**Table 1:** **Animal data for challenged and control cattle**.

Animal number	Inoculum	Genotype ***PRNP*** 211	Survival time (MPI)
1	E211K BSE-H	EK	9.8
2	E211K BSE-H	EE	18.1
3	Classical BSE	EK	25.8
4	Classical BSE	EE	25.4
5	Non-inoculated	EK	n/a
6	Non-inoculated	EE	n/a
7	Non-inoculated	EK	n/a
8	Non-inoculated	EE	n/a

*BSE, bovine spongiform encephalopathy; PRNP, prion protein gene; EE, glutamate glutamate (wild type polymorphism); EK, glutamate lysine; MPI, months post-inoculation; n/a, not applicable*.

## Results

### Incubation Period

Survival times for BSE-C inoculated animals were similar (Table 1). For E211K BSE-H inoculated animals, the survival time for the EK211 animal, 9.8 months, was much shorter than that for the EE211 animal (18.1 months) (Table 1).

### Clinical Signs

All challenged cattle developed clinical disease with survival times of between 9.8 and 25.8 MPI (Table 1). Clinical presentation of animal #1 (*PRNP* genotype EK211, challenged with E211K BSE-H) has been reported previously (10). Briefly, early clinical signs observed at 9.4 MPI included listlessness, low head carriage, and decreased feed consumption. One week later, animal #1 was separating himself from other animals in the pen, head pressing, intermittently reluctant to rise, and ataxic when moving. He began repetitive, excessive lip licking and sham chewing behaviors that were not associated with feeding. By 9.8 MPI, animal #1 was depressed, salivating excessively, and reluctant to rise so was euthanized.

Animal #2 (EE211, E211K BSE-H) demonstrated a low head carriage, i.e., head down in non-physiologic position with drooping ears, at 14.8 MPI. By 17.7 MPI animal #2 was listless, had begun head pressing, was reluctant to move, and showed forelimb ataxia. Approximately 2 weeks later (18.1 MPI) animal #2 was observed to be pacing in circles in the pen; walking in a figure eight motion and turning only when her nose or ears came in contact with a wall or gate. She was euthanized the next day.

Animal #3 (EK211, BSE-C) was noted to be hyper-responsive to sound at 14.6 MPI but was not ataxic at this time. Inappetance was first noted at 19.4 MPI and by 25.3 MPI animal #3 displayed distinct weight loss. A slight increase in startle response was noted at 20.2 MPI but did not progress. Equivocal ataxia was observed as early as 14.8 MPI and progressed to hopping of the hind legs (23.4 MPI), goose-stepping (25.3 MPI), and difficulty rising (25.8 MPI). Animal #3 was euthanized at 25.8 MPI.

The earliest unequivocal clinical signs displayed by animal #4 (EE211, BSE-C) were an increase in startle response and decreased appetite at 19 MPI. Hyperreactivity increased and appetite decreased over the following 3 months and at 22 MPI animal #4 began scratching and pressing her head against the wall or gate. Proprioceptive deficits and ataxia were noted at 24.3 MPI and distinct weight loss and abnormal licking and chewing behaviors at 25.3 MPI. One week later (25.4 MPI), animal #4 developed a head tremor and twitching, had difficulty rising and moved with a stumbling gait for a brief time after rising, and displayed hind-limb ataxia and staggering when moving and turning. Animal #4 was euthanized at that time.

### Electroretinography

Electroretinography is used to objectively measure retinal function by measuring several attributes of the resulting waveform. Here, electroretinography was used to assess any effects of host or inoculum genotype on retinal function during BSE incubation.

**Figure 1:**
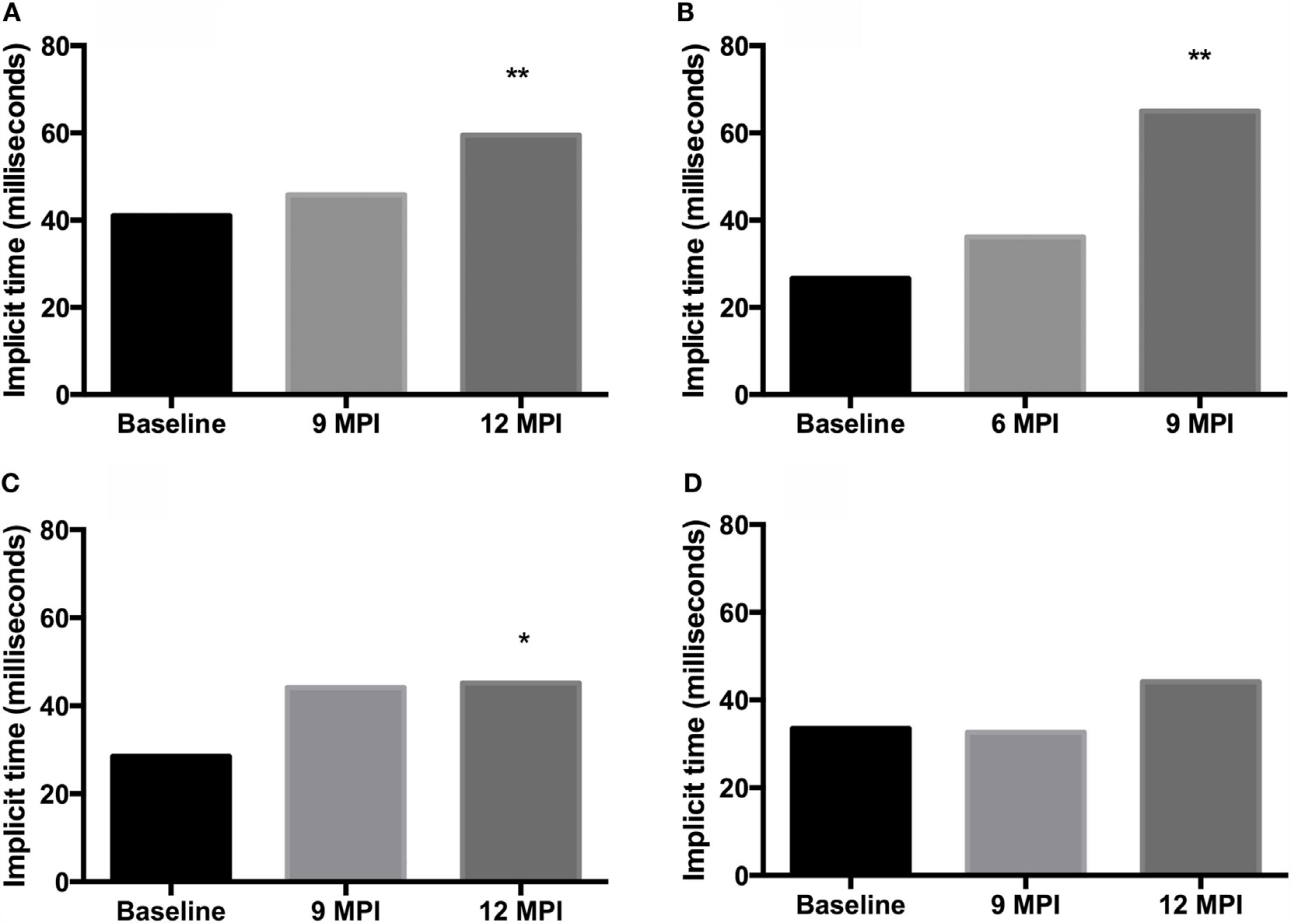
**B-wave implicit times, measured using electroretinography, for challenged cattle**. **(A,B)** E211K BSE-H challenged cattle, **(A)** *PRNP* genotype EE211and **(B)** *PRNP* genotype EK211. **(C,D)** classical BSE challenged cattle, **(C)** *PRNP* genotype EE211and **(D)** *PRNP* genotype EK211. MPI, months post inoculation. Stars indicate statistically significant differences between baseline data (collected 1 day prior to inoculation) and data collected at final examination: *P < 0.05, **P < 0.001.

Analysis of b-wave implicit times show a prolongation of the b-wave implicit time in all animals between pre-inoculation (baseline) testing and testing at clinical end-point or 12 MPI. This prolongation was statistically significant in both E211K BSE-H inoculated cattle and the EE211 BSE-C inoculated animal, but not in the EK211 BSE-C inoculated animal (Table 2, Figure 1).

**Table 2:** **Electroretinography (b-wave implicit time) and optical coherence tomography (retinal thickness) results for challenged and control cattle**.

	B-wave implicit time (milliseconds ± SEM)				Retinal thickness (μm ± SEM)			
Case number	Pre-inoculation	6 MPI	9 MPI	12 MPI	Pre-inoculation	12 MPI	Clinical	Control animals
1	26.7 (2.0)	36.7 (1.8)	65.0 (1.2)**	n/a	371 (27)	n/a	275 (6)	
2	41.0 (0.9)	42.5 (0.5)	45.8 (4.7)	59.5 (2.5)**	287.6 (2.5)	259.8 (3.6)	270.4 (3.8)	
3	33.5 (0.6)	41.5 (2.8)	32.7 (0.7)	44.2 (5.5)	325 (8.1)	270.7 (2.4)	275.1 (1.5)	
4	28.5 (9.0)	25.8 (2.4)	44.7 (1.8)	45.2 (5.4)*	274.8 (12.3)	265.3 (3.1)	250.7 (2.0)	
5								302.1 (3.9)
6								308.3 (5.9)
7								309.6 (3.3)
8								289.9 (2.9)

*SEM, standard error of the mean of three technical replicates (B-wave implicit time) or 10 technical replicates (retinal thickness); MPI, months post-inoculation*.

*P < 0.05.

**P < 0.001.

### Optical Coherence Tomography

To investigate the effect of host or inoculum genotype on retinal thickness during BSE incubation OCT was used to measure retinal thickness *in vivo*. Retinal thickness of experimental animals prior to inoculation was not significantly different than the control group values (Mann–Whitney test, *P* > 0.99) (Table 2). Retinal thickness in all BSE challenged animals decreased between pre-inoculation testing and testing at 12 MPI, which was independent of incubation time.

One animal (case #1, EK211, E211K BSE-H) developed clinical disease and was necropsied at 9.8 MPI so was not included in the 12 MPI test. For animal #2 (EE211, E211K BSE-H) and animal #3 (EK211, BSE-C), retinal thickness values at 12 MPI were not significantly different to those at clinical stages (paired *t*-test, both *P* > 0.1). However, for animal #4 (EE211, BSE-C) retinal thickness at clinical stages was significantly thinner than at 12 MPI (P < 0.01). Retinal thickness of all challenged animals at clinical stages was significantly decreased compared to age-matched negative control animals (*P* = 0.03) (Table 2).

### Vacuolation Lesion Profiles

Some neuroanatomical areas of interest included in the Simmons et al. (23) vacuolation profiling method were not available for examination in some cattle in this study. Therefore, when comparing vacuolation lesions profiles, only the neuroanatomical areas that were available for examination in all cattle were included in the analysis, i.e., nucleus of the solitary tract, nucleus of the spinal tract of the trigeminal nerve, hypoglossal nucleus, cerebellar vermis, medial geniculate nucleus, frontal cortex, caudate nucleus, putamen, and claustrum.

**Figure 2:**
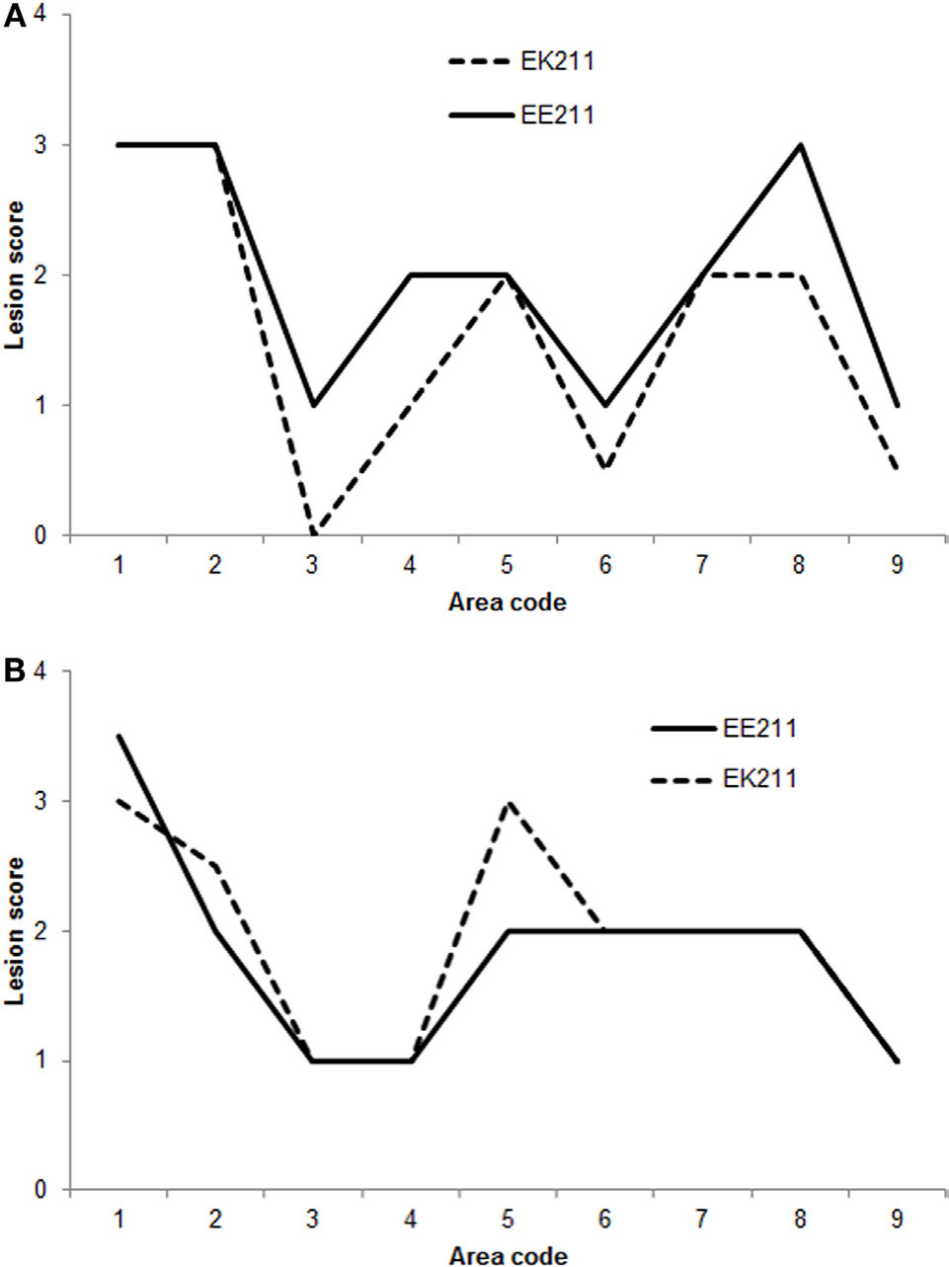
**Vacuolation lesion profiles**. **(A)** BSE-C challenged cattle. **(B)** BSE-H challenged cattle. Neuroanatomical areas examined: 1, nucleus of the solitary tract; 2, nucleus of the spinal tract of the trigeminal nerve; 3, hypoglossal nucleus; 4, cerebellar vermis; 5, medial geniculate nucleus; 6, frontal cortex; 7, caudate nucleus; 8, putamen; 9, claustrum.

The vacuolation profiles for BSE-C inoculated cattle were very similar (Figure 2A). The vacuolation profiles for both E211K BSE-H challenged animals were also broadly similar (Figure 2B). Compared to BSE-C challenged animals, BSE-H challenged animals showed more severe vacuolation at the medulla at the level of the obex (nucleus of the solitary tract and hypoglossal nucleus), and in the frontal cortex.

### Immunohistochemistry

The brain, spinal cord (cervical, thoracic, and lumbar), and retina were positive in all cattle. The trigeminal ganglion was positive in the wild-type BSE-C challenged animal only. PrP^Sc^ was not present in any of the other tissues examined from any of the cattle.

### PrP^Sc^ Immunoreactivity in the Retina

**Figure 3:**
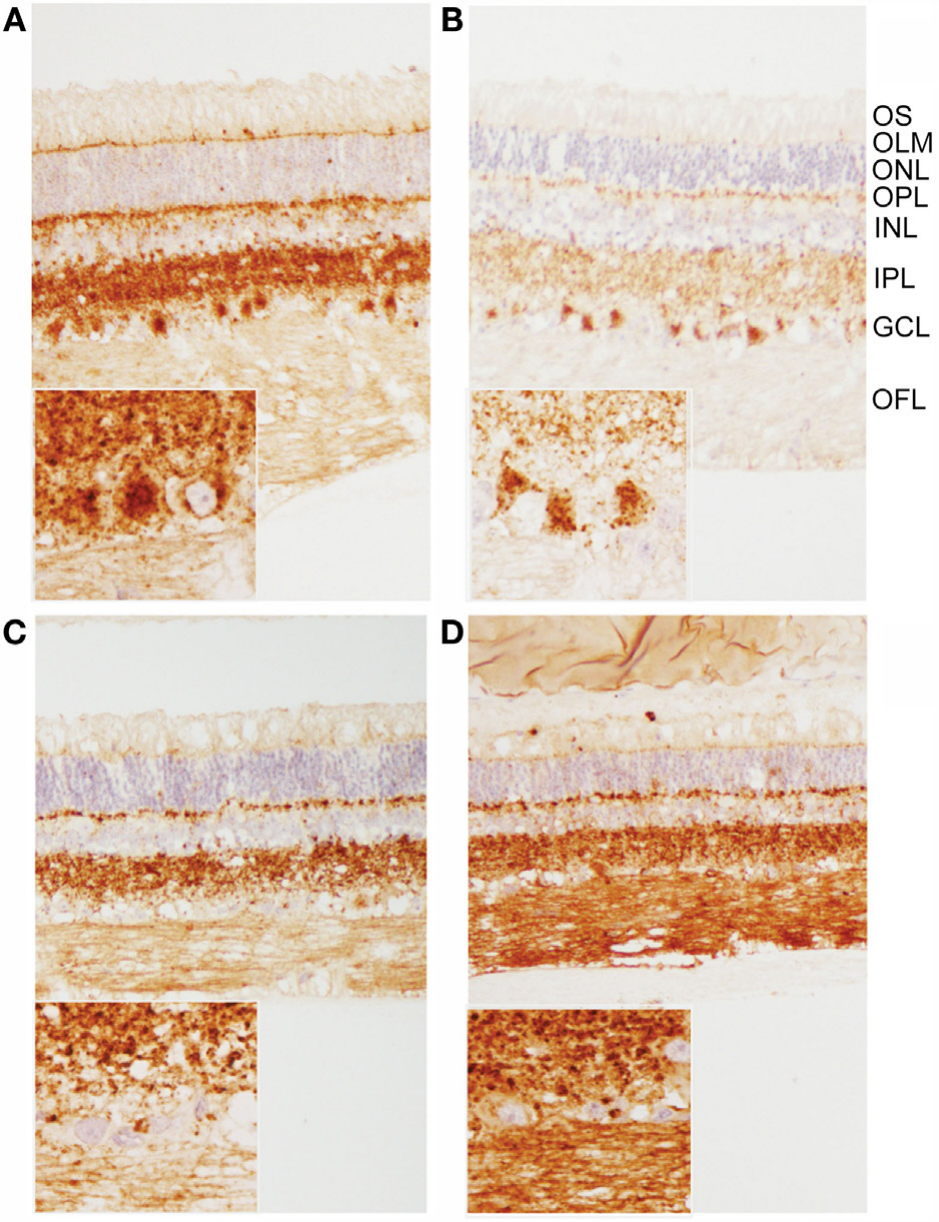
**Patterns of PrP^Sc^ immunoreactivity in the retina**. **(A,B)** Prominent PrP^Sc^ immunoreactivity in retinal ganglion cells of E211K BSE-H challenged **(A)** EK211 and **(B)** EE211 cattle. Original magnification 10×. *Inset*: higher magnification view of ganglion cell immunoreactivity. Original magnification 40×. **(C,D)** PrP^Sc^ immunoreactivity in retinal ganglion cells is much less prominent in BSE-C challenged **(C)** EK211 and **(D)** EE211 cattle. Original magnification 10×. *Inset*: higher magnification view of ganglion cell immunoreactivity. Original magnification 40×. Abbreviations, retinal cell layers: OFL, optic fiber layer; GCL, ganglion cell layer; IPL, inner plexiform layer; INL, inner nuclear layer; OPL, outer plexiform layer; ONL, outer nuclear layer; OLM, outer limiting membrane; OS, outer segments. Monoclonal anti-prion protein antibody F99/97.6.1.

When using both the R145 and 99/97 anti-PrP antibodies, immunoreactivity was observed in the inner and outer plexiform (synaptic) layers in all animals (Figure 3). However, PrP^Sc^ immunoreactivity was different in the retinas of animals inoculated with classical BSE compared to animals inoculated with E211K BSE-H. Animals inoculated with E211K BSE-H had increased accumulation of PrP^Sc^ in cell bodies of retinal ganglion cells and in the outer limiting membrane. Appreciable PrP^Sc^ immunoreactivity also was detected in the optic nerves of animals inoculated with E211K BSE-H, but not with animals inoculated with classical BSE.

### GFAP Immunoreactivity in the Retina

GFAP immunoreactivity was observed in the optic nerve and optic fiber layer in astrocytes and in Müller glia endfeet. GFAP immunoreactivity was also observed throughout Müller glia in processes spanning the retina and terminating in the outer limiting membrane. There was no appreciable difference in GFAP immunoreactivity between any of the animals (data not shown).

### PrP^Sc^ Immunoreactivity in the Brain

There was strong immunolabeling throughout the brain of the wild-type BSE-C animal (#4), including granular, linear, aggregated, intraneuronal, and stellate labeling types. There was moderately strong immunolabeling of the neocortex with prominent stellate and intra-glial labeling and less granular and intraneuronal labeling compared to the diencephalon (thalamus and hypothalamus), mesencephalon (midbrain) and rhombencephalon (medulla, pons, cerebellum). In the cerebellum, prominent stellate and linear labeling types were seen in the molecular layer, and fine granular to aggregated immunolabeling in the granular layer (Figure S1 in Supplementary Material). Compared to the wild-type BSE-C animal, the magnitude of immunolabeling was decreased in the EK211 animal (#3), particularly in the lenticular nuclei and neocortex. In addition, granular and stellate labeling types were relatively less prominent, and neocortical labeling was less severe and comprised mostly stellate labeling with mild granular labeling. Only very mild fine granular immunolabeling was present in the cerebellar cortex, although moderate granular, linear, and intraneuronal labeling was observed in the cerebellar nuclei.

**Figure 4:**
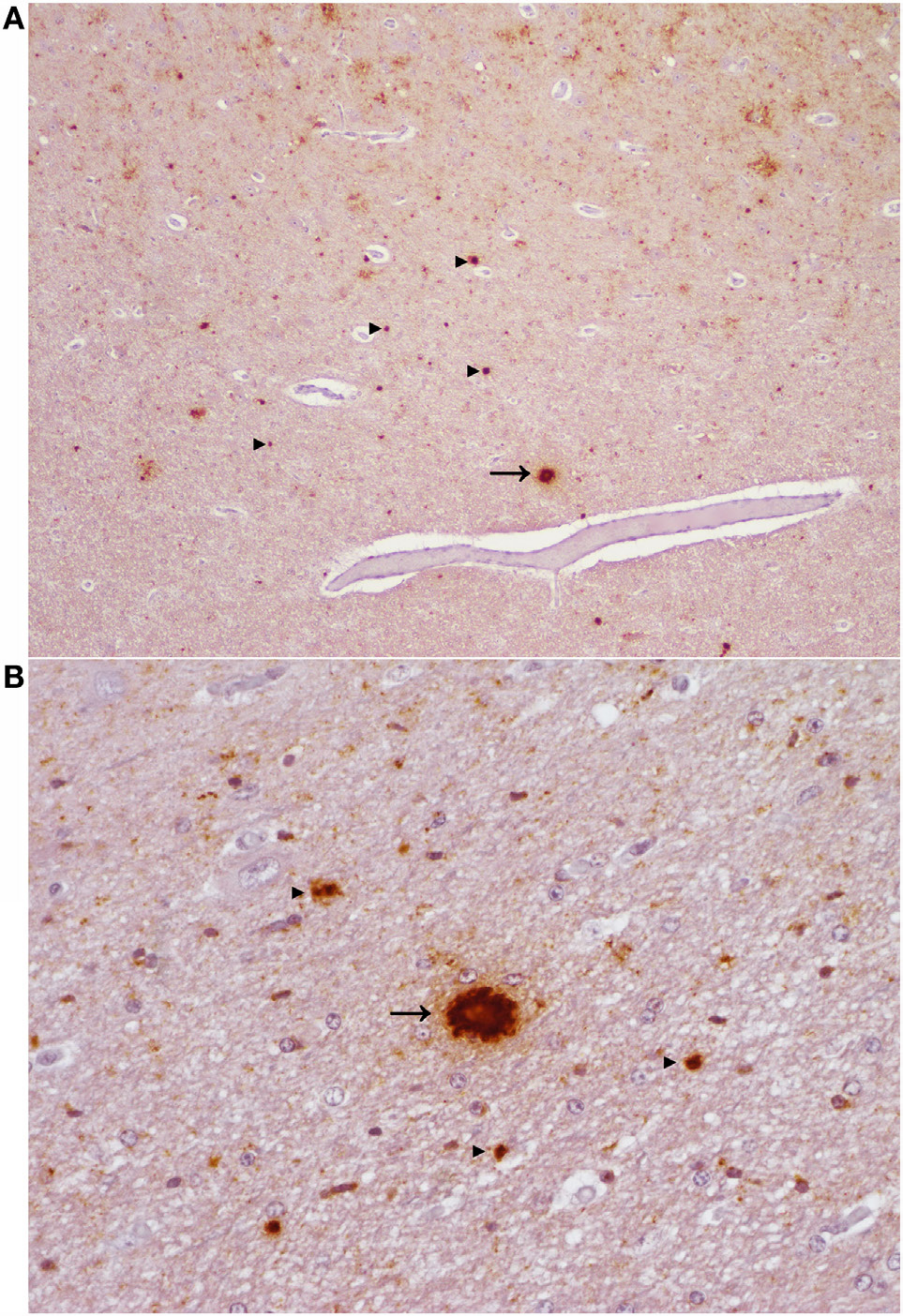
**Aggregated and plaque-like deposits observed in the E211K BSE-H challenged wild type (EE211) animal**. **(A)** Neocortex. Original magnification 5×. **(B)** Thalamus. Original magnification 20×. Arrow heads ►, aggregated PrP^Sc^ deposits. Arrow →, plaque-like PrP^Sc^ deposit. Note the lucent central area (core) which is present in the plaque-like deposits but not in the aggregated deposits.

H-type BSE challenged cattle demonstrated very little (#2 EE211) or no (#1 EK211) membrane associated labeling (linear, perineuronal) in the rhombencephalon. Small aggregates were present throughout the brain in animal #2, but only from the mesencephalon (especially in the substantia nigra) and rostrally in animal #1. The most striking feature of immunolabeling in animal #2 was large aggregates and plaque-like deposits in the diencephalon and rostrally; these deposits were particularly prominent in the neocortical white matter (Figure 4). In both BSE-H challenged cattle stellate labeling was only observed in the lenticular nuclei and neocortex. Patterns of immunolabeling in the cerebellum were broadly similar but with subtle differences: linear labeling was present in the molecular layer of animal #2, and granular labeling in the granular layer was more severe in animal #1.

### PrP^Sc^ Lesion Profiles

**Figure 5:**
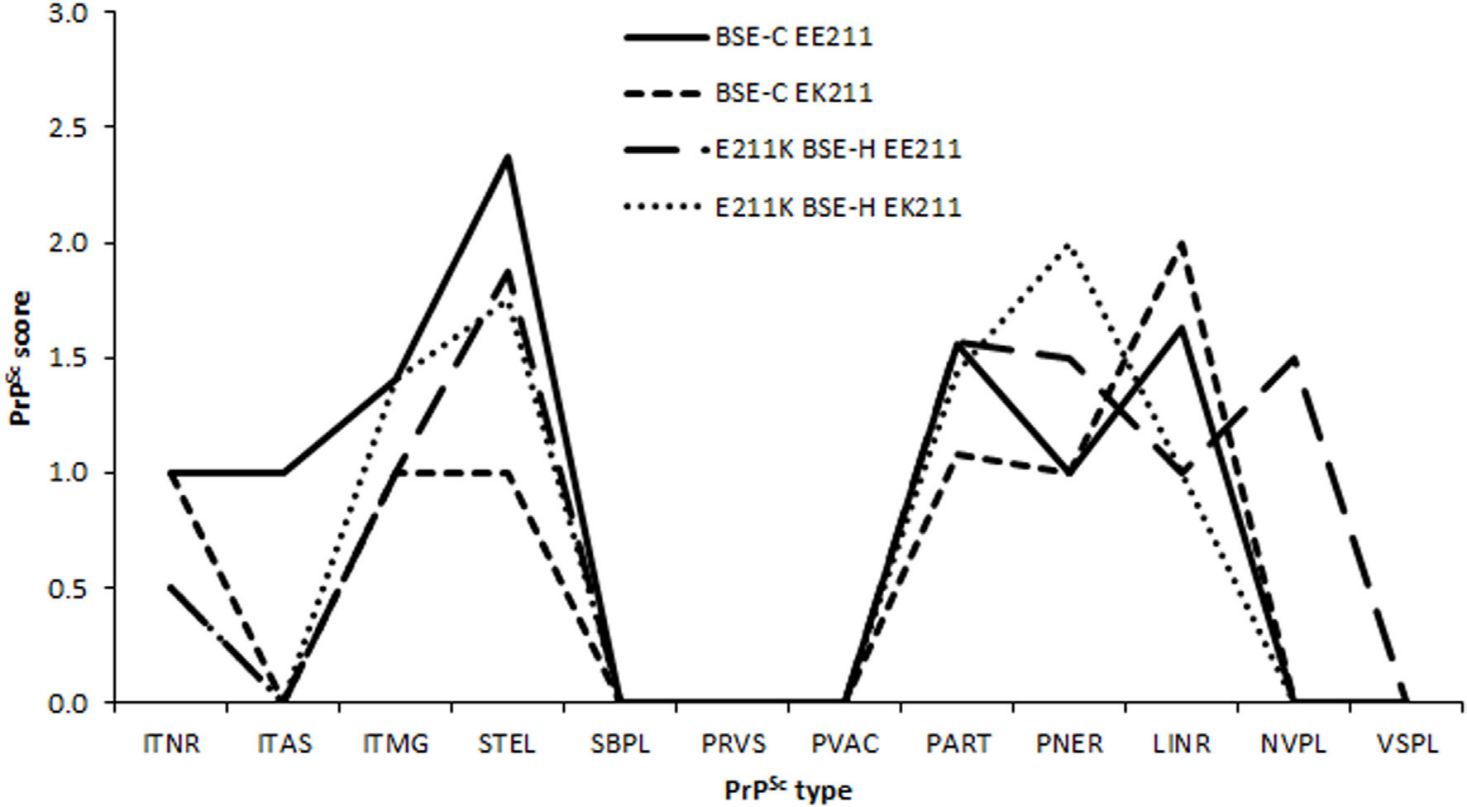
**PrP^Sc^ lesion profiles**. Legend names indicate challenge inocula and *PRNP* 211 genotype. PrP^Sc^ morphological immunolabeling types: ITNR, intraneuronal; ITAS, intra-astrocytic; ITMG, intramicroglial; STEL, stellate; SBPL, subpial; PRVS, perivascular; PVAC, perivacuolar; PART, particulate/coalescing; PNER, perineuronal; LINR, linear; NVPL, non-vascular plaques; VSPL, vascular plaques.

Due to differences in neuroanatomical areas sampled, some features were not present in all cases. Therefore, the immunolabeling types associated with these features (ependymal and subependymal) were omitted from the final comparison of PrP^Sc^ profiles. PrP^Sc^ profiles assessed at the level of the basal nuclei were broadly similar for BSE-H challenged cattle and BSE-C challenged cattle (Figure 5), although there were a number of points of difference. For BSE-C challenged cattle, the EE211 animal had a more intra-astrocytic and stellate labeling than the EK211 animal. For BSE-H challenged cattle, the EE211 animal had prominent non-vascular plaque-like deposits, particularly in the white matter, that were not observed in the EK211 animal or BSE-C challenged cattle.

### Molecular Characterization

**Figure 6:**
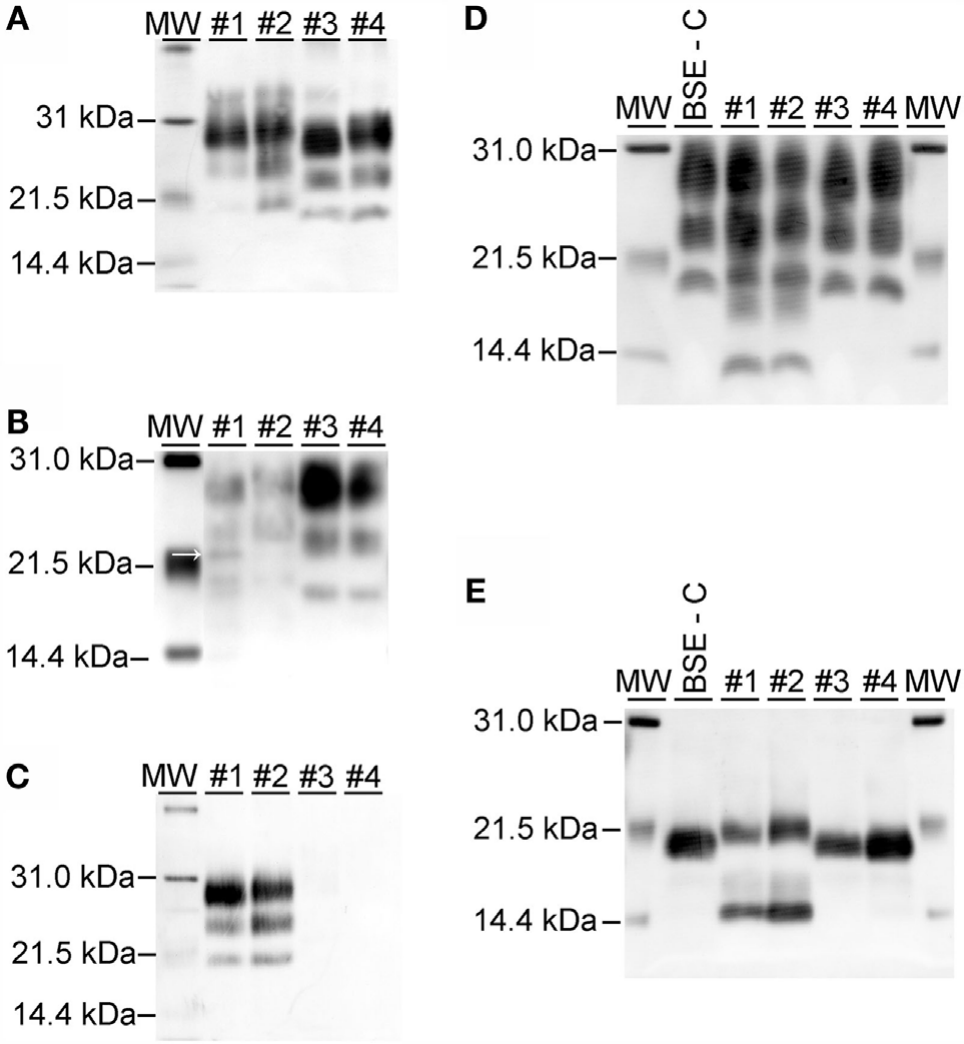
**Western blot analysis using a panel of monoclonal antibodies**. **(A)** Analysis of brainstem samples using monoclonal antibody (mAb) 6H4. The unglycosylated (lowest) band migrates higher in samples from E211K BSE-H challenged cattle (#1 and #2, *PRNP* genotypes EK211 and EE211, respectively) than in BSE-C challenged cattle (#3 and #4, *PRNP* genotypes EK211 and EE211, respectively). The diglycosylated bands are more prominent than the unglycosylated or monoglycosylated bands. **(B)** Analysis of cerebellum samples using mAb 6H4. Note the presence of an additional band at approximately 23 kDa in animal #1 (arrow). **(C)** Analysis using monoclonal antibody P4. Samples from BSE-H challenged cattle (#1 and #2) show the characteristic 3-band profile while no detectable PrP^Sc^ is observed for BSE-C samples (#3 and #4). **(D)** Analysis using mAb SAF-84. Note the presence of a 4th band at ~14 kDa in samples from BSE-H challenged cattle (#1 and #2) that is not present in BSE-C challenged cattle (#3 and #4). **(E)** Analysis using mAb SAF-84 after PNGase F treatment. There is an unglycosylated fragment at ~20 kDa, which is present in all cattle. In addition, samples from BSE-H challenged cattle present a second band at ~14 kDa. Samples were loaded at 0.5–1.0 mg equivalent of brain tissue per lane. Molecular weight standards (MW) flank the blots and the molecular weight in kiloDalton is indicated to the left of the blot.

On western blots labeled with mAb 6H4 (Figure 6A), brainstem samples from BSE-C and E211K BSE-H challenged cattle showed the characteristic three-band pattern profile of PrP^Sc^. In both BSE-C and E211K BSE-H challenged cattle, the signal for the diglycosylated band was stronger than the unglycosylated or monoglycosylated bands. Migration of the unglycosylated band of E211K BSE-H samples was higher than BSE-C samples. When cerebellum samples were examined using mAb 6H4, a fourth band migrating at ~23 kDa was present in the cerebellum from animal #1 (EK211, E211K BSE-H) (Figure 6B, arrow). This additional band was not present in cerebellum samples from other cattle in this study.

On western blots of brainstem labeled with mAb P4 (Figure 6C), BSE-H samples showed the characteristic three-band pattern profile, while no detectable PrP^Sc^ was observed for BSE-C samples. Using mAb SAF-84 (Figure 6D), samples from both BSE-C and BSE-H challenged cattle showed the characteristic three-band pattern profile of PrP^Sc^. In BSE-H cases, there is a fourth band at 14 kDa (Figure 6D) that also was present following deglycosylation (Figure 6E).

**Figure 7:**
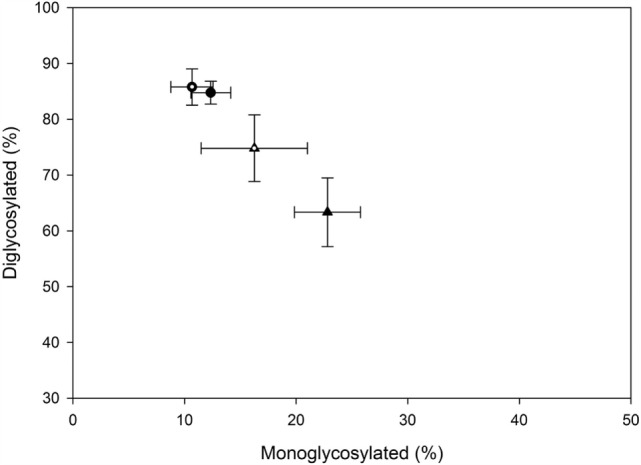
**Relative amounts of mono- and diglycosylated PrP^Sc^ in brain homogenates from challenged cattle**. Triangles represent BSE-H challenged cattle, circles represent BSE-C challenged cattle. Open triangle and open circle are cattle with the EK211 genotype. Closed triangle and closed circle are cattle with the EE211 genotype. Error bars represent the SE of the mean of seven technical replicates.

The majority of the PrP^Sc^ in all cattle was diglycosylated (average 78%), with smaller amounts of the monoglycosylated (15%) and unglycosylated (7%) PrP^Sc^. When the percentage of diglycosylated to monoglycosylated PrP^Sc^ was plotted, BSE-C challenged cattle were found to be similar to each other and different to BSE-H challenged cattle (Figure 7).

### IDEXX ELISA-Based PrP^Sc^ Stability Assays

To test for biophysical differences between PrP^Sc^ of a particular combination of inoculum and genotype, stability was determined by unfolding in GdnHCl. In a previous study, PrP^Sc^ from animal #1 (animal #25 in that publication) was shown to be more resistant to denaturation than PrP^Sc^ from animal #4 (animal #26) consistent with their assignments as BSE-H and BSE-C, respectively, by western blot (30). Stability curves for an E211K BSE-H animal were consistent with the stability curves for multiple non-E211K BSE-H animals (30).

**Figure 8:**
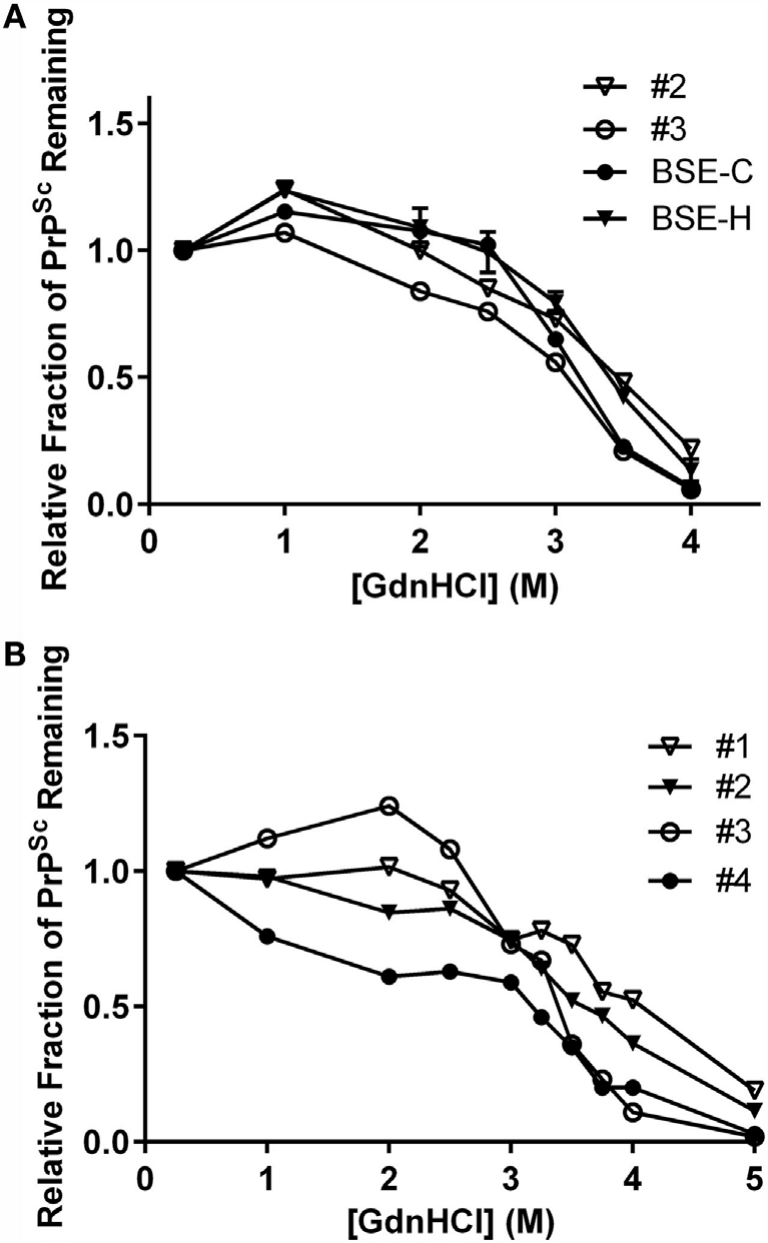
**Stability properties of BSE isolates**. **(A)** Comparison of PrP^Sc^ stabilities of heterologous inoculum-host genotype isolates. Animal #2 (*PRNP* EE211 challenged with E211K BSE-H), BSE-H (non-E211K H-type BSE), animal #3 (EK211 challenged with BSE-C), BSE-C (classical (E211E) BSE). Curves for animals #2 and #3 reflect average values of five technical replicates for each animal. Curves for BSE-C and BSE-H reflect average values of at least three biological replicates, with error bars on each of these two curves representing SE of the mean. **(B)** Stability properties of BSE isolates from challenged cattle. Animal #1 (EK211 challenged with E211K BSE-H), animal #2 (EE211 challenged with E211K BSE-H), animal #3 (EK211 challenged with BSE-C), animal #4 (EE211 challenged with BSE-C). Curves for all animals reflect average values of 3–5 technical replicates. GdnHCl stability curves in **(A,B)** were plotted based on normalization to the 0.25 M treatment point.

To expand this analysis, we characterized the GdnHCl stability profiles of PrP^Sc^ from animal #2 (EE211, E211K BSE-H) and animal #3 (EK211, BSE-C). The animal #3 sample exhibited lower stability [lower (GdnHCl)_1/2_ value] with a profile consistent with the BSE-C control (Figure 8A, circles). The animal #2 sample exhibited higher stability [higher (GdnHCl)_1/2_ value], with a profile consistent with the BSE-H control (Figure 8A, triangles).

When we compared the stability profiles for all four animals in this study (Figure 8B), curves from animals #1 and #2 (E211K BSE-H challenged cattle) generally cluster in the denaturation part of the curve, as do curves #3 and #4 (BSE-C challenged cattle). Variation in the initial region of the curve has previously been observed in non-proteinase K processed livestock samples (29, 30).

Results of GdnHCl stability curves support the findings of the western blotting on these brain homogenates. That is, samples with a western blot pattern characteristic of BSE-C also produce a GdnHCl stability profile (i.e., lower stability) consistent with BSE-C, regardless of *PRNP* genotype at codon 211. Similarly, samples from EK211 or wild-type cattle with a western blot pattern characteristic of BSE-H produce a GdnHCl stability profile (i.e., higher stability) consistent with BSE-H.

## Discussion

In this study, we investigated the role of the E211K polymorphism and its interaction with disease phenotype in animals inoculated with classical BSE or BSE-H from an animal with the E211K polymorphism (E211K BSE-H). The results presented here extend on previous published results of challenge of an EK211 calf with E211K BSE-H (10) by including data from the challenge of wild-type cattle with E211K BSE-H, and challenge of EK211 and wild-type (EE211) cattle with BSE-C. Overall, inoculum strain had a greater effect on disease phenotype than host genotype, although some differences were observed between cattle challenged with the same inoculum, in particular among the E211K BSE-H challenged cattle. However, due to the small number of animals in this study, biological variation between individual animals may have contributed to the observed differences.

The phenotype of BSE-C was similar in EK211 and wild-type cattle with regard to almost all parameters tested. For example, the incubation periods (31, 32) (Hawkins, *unpublished data*) and vacuolation profiles (33, 34) of both BSE-C challenged animals were similar to each other, and consistent with previous studies of cattle challenged with classical BSE via the intracranial route. These results are consistent with the observation that BSE-C is caused by a single prion strain with reliably consistent biological characteristics (35, 36).

One point of difference was that the wild type, but not the EK211 animal, developed abnormal licking and chewing behaviors in the clinical phase of disease. These behaviors were similar to those observed in the EK211 animal challenged with E211K BSE-H (10). The lack of any clear inoculum or genotype association with the abnormal licking and chewing behaviors suggests that it is possible that they are part of the spectrum of biological variation in the clinical presentation of BSE. A second point of difference was that the EE211 BSE-C challenged animal developed statistically significant changes in retinal function and thickness that were not as severe as in the EK211 BSE-C challenged animal.

In contrast to the relatively homogeneous phenotype observed in BSE-C challenged cattle, a number of striking differences were observed between the EK211 and wild-type cattle challenged with E211K BSE-H.

The incubation time for the wild-type animal (18 MPI) was similar to that reported for cattle challenged IC with BSE-H isolates from France (16.5–21 MPI) (37, 38), Canada (15–16 MPI) (34), or Germany (14–16 MPI) (39). The clinical duration of the wild-type animal (3.3 months) falls within the average clinical duration for BSE-H of 2–4.5 months reported in previous studies (34, 37–39). Interestingly, the incubation period for the EK211 animal (9.8 MPI) was just 55% of that of the wild-type animal challenged with the same inoculum and the clinical duration (2 weeks) was also very short. This supports our hypothesis that cattle with the E211K polymorphism are predisposed to a rapid onset of BSE-H when exposed (10).

Prominent large aggregates and plaque-like accumulations of PrP^Sc^ were observed in the wild-type animal that is consistent with previous reports for BSE-H challenged cattle (34, 37). These immunolabeling types were not observed in the E211K animal (or BSE-C challenged cattle). Both E211K BSE-H challenged cattle showed characteristic PrP^Sc^ accumulation in retinal ganglion cells and a significant increase in b-wave implicit time, significant retinal thinning compared to age-matched controls, as has been reported previously for E211K and wild-type BSE-H cases (10, 40).

The K211 allele did not affect the molecular profile of PrP^Sc^ in BSE-C challenged cattle and, thus, would not mask the presence of feedborne BSE-C. PrP^Sc^ from the brainstem at the level of the obex showed a similar western blot migration pattern in the two E211K BSE-H challenged cattle. However, the relative amounts of mono- versus diglycosylated PrP^Sc^ in these samples were different, with values for the EK211 animal lying midway between the wild-type EK211 BSE-H challenged animal and the two BSE-C challenged animals. Furthermore, on western blot of cerebellum, an additional band was observed in the EK211 animal challenged with E211K BSE-H but not the wild-type animal. These differences in the molecular characteristics appear to be genotype, rather than inoculum, driven and further work is needed to determine the molecular explanations for these observations.

Previously, we demonstrated that BSE-C and BSE-H cattle brain homogenates can be discriminated by their resistance to denaturation in GdnHCl in a whole homogenate, ELISA-based stability assay (30). Specifically, BSE-H PrP^Sc^ is more resistant to denaturation in GdnHCl than BSE-C PrP^Sc^. Data from the present study support our previous findings. In addition, stability curves for cattle that received the same inoculum were similar to each other, regardless of *PRNP* genotype, and even in the case of heterologous inoculum-host genotype combinations, the stability curve of the recipient was similar to the inoculum isolate, rather than the homologous genotype inoculum isolate.

The bovine E211K *PRNP* polymorphism is homologous to the human E200K mutation that is associated with fCJD (41). Therefore, we considered possible similarities between the epidemiology and phenotypic expression of prion disease in EK200 humans and EK211 cattle.

The human E200K mutation accounts for >70% of cases of fCJD (42), while the bovine E211K polymorphism has only been detected in a single case (8) out of the ~60 cases of atypical BSE-H diagnosed to date (43). The E211K polymorphism is very rare in cattle; it has not been detected in cattle tested in the United States (*n* = 6062 cattle tested) (44), China (*n* = 349) (45), Poland (46), or Pakistan (*n* = 236 cattle and *n* = 281 buffalo) (47). We currently have non-inoculated heterozygous and homozygous K211 cattle under observation for the potential development of genetic BSE-H.

On western blot, an additional fragment in PrP^Sc^ from cerebellum has been reported in an E200K-129V CJD case (48). This band migrates at 17 kDa in contrast to the 23 kDa additional band observed in the cerebellum from the E211K BSE-H challenged EK211 animal in this study (10). With regard to immunohistochemistry, the characteristic stripe-like immunolabeling in the molecular layer of the cerebellar cortex reported in E200K CJD cases (4) was not observed in cattle in this study. Nor was “type III” intraneuronal immunoreactivity (small, globular, 1.5–4.0 μm diameter, darkly immunostained, intracytoplasmic PrP^Sc^ accumulations), which has been significantly associated with a subset of E200K fCJD cases (49).

Due to the rarity of the E211K polymorphism in cattle and the small number of cases of prion disease in E211K cattle available for examination at this time, further work is needed to determine the relationship between prion disease associated with the E211K bovine polymorphism and the human E200K mutation.

In this study, we have demonstrated that, in EK211 and wild-type cattle challenged with BSE-C or E211K BSE-H, inoculum strain has a greater effect on disease phenotype than host genotype. The phenotype of E211K BSE-H remains stable when transmitted to cattle without the K211 polymorphism, although a number of differences were observed between the EK211 compared to the wild-type animal. In addition, the phenotype of E211K BSE-H shows a number of features that allow it to be differentiated from BSE-C in both wild-type and heterozygous animals.

## Author Contributions

JG and EN conceived of the study. SM, MG, JS, and JG performed animal studies, microscopic examination of tissues, interpretation of data, statistical analyses, and drafted the manuscript. CV and EN performed and interpreted PrP^Sc^ stability assays. All authors have read and approved the final draft of this manuscript.

## Conflict of Interest Statement

The authors declare that the research was conducted in the absence of any commercial or financial relationships that could be construed as a potential conflict of interest.
