# Supplementary figures and images for: A Comparison of Classical and H-Type Bovine Spongiform Encephalopathy Associated with E211K Prion Protein Polymorphism in Wild-Type and EK211 Cattle Following Intracranial Inoculation
Source: Front Vet Sci. 2016 Sep 15;3:78. doi: 10.3389/fvets.2016.00078 (PMC5023952; doi:10.3389/fvets.2016.00078)

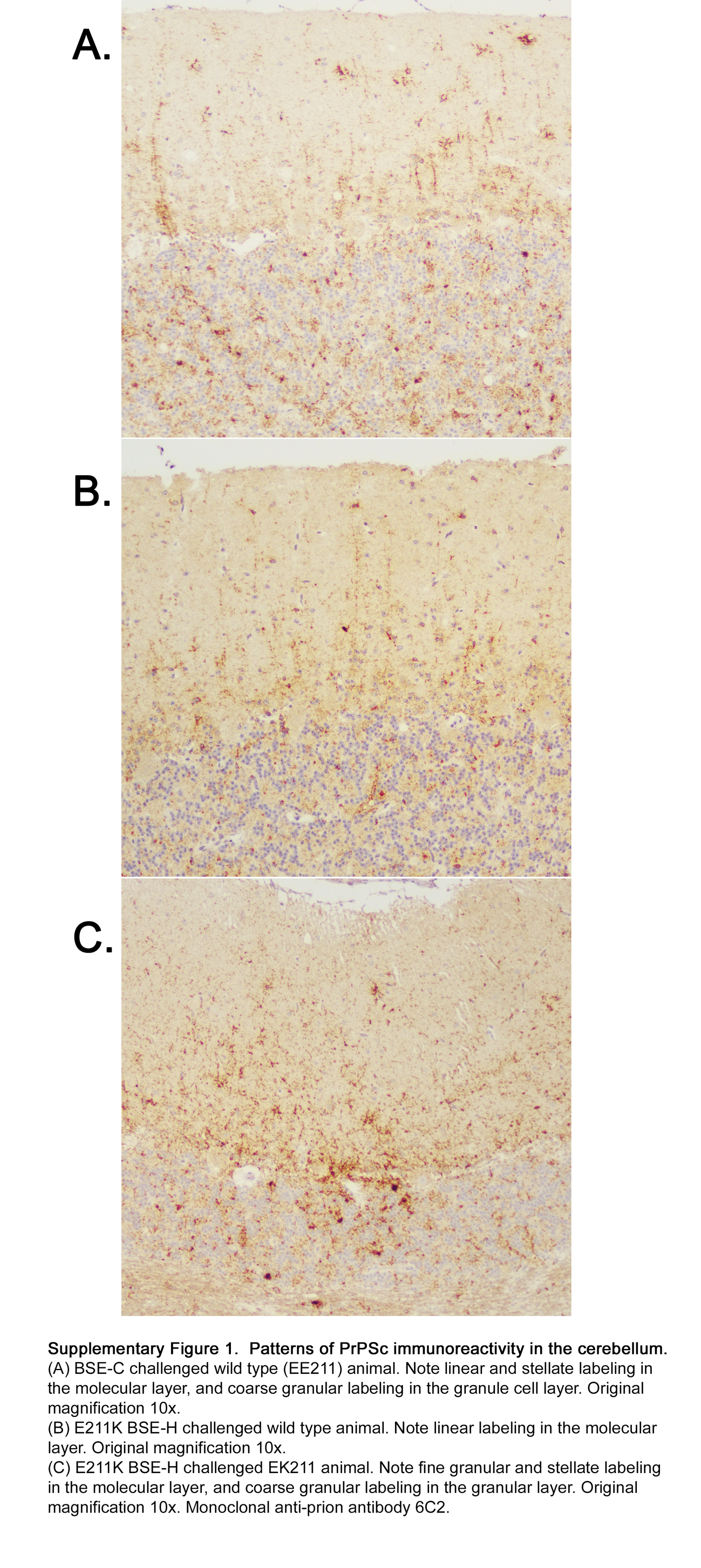

Supplement: Supplementary file 1 [file Image_1.TIF]
